# Plerixafor in non-Hodgkin’s lymphoma patients: a German analysis of time, effort and costs

**DOI:** 10.1038/s41409-018-0228-z

**Published:** 2018-05-24

**Authors:** Kai Hübel, H. Ostermann, Bertram Glaß, Richard Noppeney, Florian Kron, Anna Kron, Gary Milkovich, Mohamad Mohty

**Affiliations:** 1 Department of Internal Medicine, University Hospital of Cologne, Cologne, Germany; 2 Department of Internal Medicine III, University of Munich, Munich, Germany; 3 Department of Hematology, Oncology and Cancer Immunology, Helios-Klinikum Berlin-Buch, Berlin, Germany; 4 Hematology, University Hospital Essen, Essen, Germany; 5 Department of Internal Medicine, Center for Integrated Oncology, University Hospital of Cologne, Cologne, Germany; 6 Department of Internal Medicine, Center for Integrated Oncology, University Hospital of Cologne, Cologne, Germany; 7 RJM Group, LLC, 13028 Smoketown Road, Woodbridge, VA 22192 USA; 8 Department of Haematology, Saint-Antoine Hospital, Paris, France

**Keywords:** Stem-cell therapies, Non-hodgkin lymphoma

## Abstract

Mobilization and collection of peripheral blood stem cells is part of the standard treatment procedure for non-Hodgkin’s lymphoma patients eligible for high-dose chemotherapy with autologous stem cell transplantation. Mobilization is usually achieved with chemotherapy and/or cytokines, but plerixafor might be added in case of poor mobilization. Due to the high cost several institutions have developed their own management pathway to optimize use of plerixafor. Such models are however rarely generalizable; in a multi-center, European, non-interventional study, evaluating the impact of plerixafor in poor mobilizers, country specific differences in patient treatment and cost structure were obvious. For German centers, there was a non-significant reduction in the number of apheresis sessions carried out and in apheresis costs. In contrast to other European countries the majority of German Plerixafor patients were very poor mobilizing patients with initial CD34+ cell count ≤ 10/µl (40/51). In this group the number of apheresis sessions decreased from 2.1 to 1.6 sessions per patient (*p* = 0.01) and costs decreased from €6246 to €4758 (*p* = 0.01). Our results show that preemptive plerixafor use has a strong effect in poor mobilizers with an initial CD34+ cell count ≤ 10 cells/µl.

## Introduction

High-dose chemotherapy (HDC) with autologous stem cell transplantation (ASCT) has become the standard of care for patients with relapsed and/or high-risk non-Hodgkin’s lymphoma (NHL) ever since clinical trials have shown a benefit over standard chemotherapy in terms of progression-free and overall survival [1–4]. Collection of peripheral blood progenitor cells (PBSC) is usually carried out after induction chemotherapy and mobilization with cytokines. However, 10–25% of NHL patients fail to achieve sufficient stem cell yields to proceed to transplantation with current mobilization regimens. These patients either undergo further mobilization attempts or receive alternative treatment options, requiring additional health care resources. In addition, mobilization failure impacts treatment outcome; in a retrospective study it was found that the three-year survival rate was 33% in poor mobilizers (CD34+ < 2 × 10^6^ cells/kg) as compared to 71% in patients mobilizing adequately [5].

Improving mobilization strategies and the prediction of poor mobilizers may reduce the need for additional health care resources. Progress has been made in both directions with the discovery of risk factors associated with poor mobilization [6–8] and the introduction of plerixafor as mobilizing agent in conjunction with G-CSF with or without chemotherapy. Plerixafor antagonizes the interaction between stromal derived factor 1 and CXCR4 and by doing so interferes with homing of hematopoietic progenitor cells to the bone marrow. It was shown that plerixafor when given together with G-CSF, increased circulating PBSC several fold and could thereby rescue patients from mobilization failure when given together with conventional mobilization regimens [9].

Current guidelines by American and European societies recommend the use of plerixafor in a pre-emptive way based on CD34+ cell count or as salvage therapy in case of low apheresis yield [10, 11]. Due to the high cost of plerixafor, several institutions have published pharmacoeconomic analyses to identify the most cost-effective mobilization strategy. These studies have shown that plerixafor used in a pre-emptive way in patients identified as poor mobilizers decreased mobilization failure rate and increased the number of patients proceeding to transplantation at an acceptable additional cost [12–15]. The best predictor of poor mobilization was shown to be the surrogate marker CD34+ for circulating stem cells [16]. Patients with a pre-apheresis CD34+ cell count < 20 cell/µl are usually referred to as poor mobilizers, however, cut-off values may vary among institutions and countries as a consequence of different patient population, mobilization goals, chemotherapy regimens, and reimbursement policies. We recently carried out a non-interventional study to evaluate the impact of plerixafor on apheresis in poor mobilizers, i.e., patients with a CD34+ cell count < 20 cell/µl. The study was carried out at 10 centers in France, Germany, and Italy. Overall, plerixafor reduced the mean number of apheresis sessions needed per patient, time spent on apheresis, and costs related to apheresis [17].

In Germany, these reductions were more modest. Therefore, we conducted a separate analysis on the German data to obtain an undiluted picture of the changes brought by plerixafor in German clinical practice.

## Methods

This is a country-specific analysis of an international, multicenter, retrospective and prospective observational study. The study design was as described elsewhere [17]. Briefly, NHL patients undergoing ASCT and qualifying as poor mobilizers, i.e., CD34+ count < 20 × 10^6^ cells/kg after mobilization, were enrolled from two-time periods: prior to approval of plerixafor (July 2009, pre-plerixafor era) and after approval of plerixafor (plerixafor era). The study was conducted at ten European centers, four of them being in Germany. Patients were eligible if 18 years and older with a diagnosis of NHL.

The study protocol was approved by the central and local ethics committees, with the ethics committee of the University of Cologne acting as the central ethics committee. No informed consent was required for this retrospective study. This study was registered with ClinicalTrials.gov, number NCT02287012.

### Data collection

For the retrospective part, patient records were analyzed from 1 June 2007 to 1 June 2009 for the pre-plerixafor era and from 1 July 2010 to 1 July 2012 for the plerixafor era. At each center patients fulfilling inclusion criteria were sequentially included until a number of 20 patients was reached or all records were exhausted.

Each plerixafor era patient was matched on a 1:1 basis to a pre-plerixafor era patient, based on CD34+ target levels. Enrollment continued until a single CD34+ target level match was found for each plerixafor era patient or the pool of pre-plerixafor patients was exhausted. If no CD34+ target level match was found, the plerixafor patient was excluded.

The following data were extracted: baseline characteristics, diagnosis (NHL subtype), number of mobilization visits and mobilization agents, adverse events, number and duration of apheresis sessions, total CD34+ cells collected and transplanted, transplantation outcome, and costs associated with mobilization, apheresis, and cryopreservation. Costs were estimated through interviews with local hospital administration at one study site in Cologne. Items included were (1) clinical chemistry costs prior to apheresis for CD34 levels, (2) day hospital stay for apheresis (3–4 h), (3) fixed costs for apheresis including medical supplies, solutes, harvest Kit, and overhead, (4) manipulation, cell engineering, materials costs, personal costs and storage for one bag of collected stem cells, (5) thawing costs for one bag of stem cells infused, including medical supplies, equipment amortization, personal costs (Supplementary information [SI] Table 1).

**Table 1:** Baseline characteristics

	Pre-plerixafor era (*n* = 39)		Plerixafor era (*n* = 51)		*p*<
Age (years); mean (SD)	56	(12)	58	(12)	0.46^a^
Months since diagnosis					
Mean (SD)	46	(56)	26	(42)	
Median (Q1–Q3)	26	(7–53)	11	(6–38)	
Range (min–max)		(2–216)		(2–270)	0.06^b^
Gender					
Female—*N* (%)	12	(31%)	16	(31%)	
Male—*N* (%)	27	(69%)	35	(69%)	0.95^c^
NHL subtype					
Follic—*N* (%)	8	(21%)	9	(18%)	
Diffuse—*N* (%)	6	(15%)	17	(33%)	
Mantle—*N* (%)	12	(31%)	9	(18%)	
Other—*N* (%)	13	(33%)	13	(31%)	0.21^c^
Disease stage at diagnosis					
1—*N* (%)	5	(13%)	3	(6%)	
2—*N* (%)	6	(15%)	7	(14%)	
3—*N* (%)	8	(21%)	8	(16%)	
4—*N* (%)	19	(49%)	32	(63%)	
Unknown—*N* (%)	1	(3%)	1	(2%)	0.67^c^

^a^Student’s *t*-test

^b^Wilcoxon rank sum

^c^Chi square

Time-motion analysis was conducted on prospectively enrolled patients undergoing ASCT to validate the time spent on apheresis derived from retrospective hospital records [17]. The time spent for clinical assessment, medical record entry, management of supplies, apheresis, and other procedures, was recorded on the case report form (CRF) by the time-motion observer.

All patient data from the retrospective and prospective parts were transcribed in the CRF in an anonymous fashion according to current requirements (no patient initials and no connection table between patient number and patient file).

### Statistical analysis

The primary endpoint of this study was time and effort to mobilize patients for ASCT, using two main variables, namely mean time to perform apheresis and cost per patient inferred to the hospital.

Secondary endpoints included number of visits for mobilization purposes and number of days receiving mobilizing agents, number and duration of apheresis sessions, time from apheresis to transplant, transplant outcome, attainment of CD34+ target and days until target was met, and adverse events during mobilization.

The sample size for the original multi-center study was based on results reported by Micallef et al. for the number of days needed for mobilization and collection during the period prior to and after the introduction of plerixafor [13]. Here, a reduced number of records limited to the German sites only was considered and hence the power of detecting a difference between the two time-period was reduced (the power was 57.3% for detecting a difference of 1.4 days in mobilization and collection days assuming a one-tailed test and with *α* = 0.05).

For categorical data, differences between eras were evaluated using McNemar’s test for matched pairs. For continuous data, difference scores were calculated by subtracting the value for each plerixafor era patient from the value of his/her matched controlled. If the difference score was normally distributed, statistical significance was assessed using the paired *t*-test. If the data was not normally distributed, the Wilcoxon signed rank test was used instead. Normality of each distribution was determined using Kolmogorov-Smirnov test.

## Results

After analysis of hospital records at four German sites (University Cologne, University Essen, University Munich, Asklepios Hospital Hamburg), 90 patients were identified fulfilling the inclusion criteria. Thirty-nine were treated during the pre-plerixafor era and 51 were treated during the plerixafor era. Baseline characteristics between the two patient groups were comparable (Table 1). The initial CD34+ count was significantly higher in the pre-plerixafor than in the plerixafor era (*p* < 0.001).

Patients treated during the plerixafor showed a trend towards fewer apheresis sessions, lower total apheresis blood volume and less time spent on apheresis. These differences were not statistically significant (Table 2). A CD34+ collection yield of 2 × 10^6^ cells/kg was achieved by 34 patients (83%) treated in the pre-plerixafor period and by 46 patients (90%) in the plerixafor era (*p* = 0.74). The costs associated with apheresis decreased from €5631 to €4765 (*p* = 0.07).

**Table 2:** Apheresis activities

	Pre-plerixafor era (*n* = 39)		Plerixafor era (*n* = 51)		*p*-value
Initial peripheral CD34+ (cells/µl)					
Mean (SD)	11.7	(5.9)	7.1	(4.7)	
Median (Min; Max)	11.4	(1.0; 20.0)	6.6	(1.0; 19.8)	0.001^b^
Number of apheresis sessions					
Mean (SD)	1.9	(0.8)	1.6	(0.7)	
Median (Min; Max)	2.0	(1.0; 4.0)	2.0	(1.0; 4.0)	0.07^b^
Estimated apheresis cost (€)					
Mean (SD)	5631	(2364)	4764	(1939)	
Median (Min; Max)	5856	(2928; 11712)	5856	(2928; 11712)	0.07^b^
Total apheresis blood volume (l)					
Mean (SD)	26.6	(13.5)	22.0	(12.7)	
Median (Min; Max)	26.9	(9.9; 64.4)	21.0	(4.7; 67.0)	0.06^b^
Total minutes of apheresis					
Mean (SD)	385	(166)	332	(141)	
Median (Min; Max)	320	(135; 1000)	285	(125; 645)	0.11^b^
CD34+ cells, total (x10^6^ cells/kg)					
Mean (SD)	5.5	(3.6)	4.5	(3.1)	
Median (Min; Max)	4.8	(0.7; 16.7)	3.6	(0.8; 13.8)	0.08^b^
CD34+ cells, first apheresis (x10^6^ cells/kg)					
Mean (SD)	3.6	(3.3)	2.9	(2.5)	
Median (Min; Max)	2.0	(0.5; 12.2)	1.9	(0.7; 13.8)	0.86^b^

^b^Wilcoxon rank sum

In patients with very low initial CD34+ count (≤10 cells/µl), the mean number of apheresis sessions, total apheresis volume, and time spent on apheresis significantly decreased in the plerixafor era (Table 3). The average yield after the first apheresis session increased from 1.5 × 10^6^ cells/kg to 2.6 × 10^6^ cells/kg (*p* = 0.01). In addition, the reduction in costs associated with apheresis was more marked, with a decrease from €6246 in the pre-plerixafor era to €4758 in the plerixafor era (*p* < 0.01).

**Table 3:** Apheresis activities in patients with CD34+ count < 10 cells/µl

	Pre-plerixafor era (*n* = 15)		Plerixafor era (*n* = 40)		*p*-value
Initial peripheral CD34+ (cells/µl)					
Mean (SD)	5.6	(3.5)	5.1	(2.8)	
Median (Min; Max)	5.2	(1.0;10.0)	5.0	(1.0; 10.0)	0.60^b^
Number of apheresis sessions					
Mean (SD)	2.1	(0.5)	1.6	(0.7)	
Median (Min; Max)	2.0	(1.0; 3.0)	2.0	(1.0; 4.0)	0.01^b^
Estimated apheresis cost (€)					
Mean (SD)	6246	(1512)	4758	(1954)	
Median (Min; Max)	5856	(2928; 8784)	5856	(2928; 11712)	0.01^b^
Total apheresis blood volume (l)					
Mean (SD)	31.3	(12.3)	21.9	(12.9)	
Median (Min; Max)	28.6	(13.1; 64.4)	20.9	(4.7; 67.0)	0.01^b^
Total minutes of apheresis					
Mean (SD)	429	(157)	338	(139)	
Median (Min; Max)	400	(265; 759)	288	(150; 645)	0.04^b^
CD34+ cells, total (x10^6^ cells/kg)					
Mean (SD)	3.2	(1.9)	4.3	(2.8)	
Median (Min; Max)	3.3	(0.7-5.8)	3.6	(0.8; 12.9)	0.30^b^
CD34+ cells, first apheresis (x10^6^ cells/kg)					
Mean (SD)	1.5	(0.8)	2.6	(1.6)	
Median (Min; Max)	1.5	(0.5; 3.0)	2.0	(0.7; 8.1)	0.01^b^

^b^Wilcoxon rank sum

In the plerixafor era more patients proceeded to engraftment, namely 44 out 51 patients (86%) as compared to 24 out of 39 patients (62%) in the pre-plerixafor era (*p* = 0.01; Table 4). There was no significant difference in the proportion of patients achieving platelet and neutrophil engraftment nor in the time to engraftment.

**Table 4:** Transplantation and engraftment

	Pre-plerixafor era (*n* = 39)	Plerixafor era (*n* = 51)	*p*-value
Patients proceeding to SCT; *n* (%)	25/39 (62%)	44/51 (86%)	0.01^c^
SCT patients achieving platelet engraftment	16/24 (67%)	28/44 (64%)	0.80^c^
SCT patients achieving neutrophil engraftment	22/24 (92%)	39/44 (89%)	0.69^c^
Days—Apheresis to SCT			
*n*	24	44	
Mean (SD)	59 (40)	58 (46)	
Median (Min; Max)	47 (9; 208)	42 (22; 286)	0.41^b^
Days—SCT to platelet engraftment			
*n*	16	28	
Mean (SD)	18 (19)	17 (12)	
Median (Min; Max)	15 (6; 45)	14 (1; 15)	0.71^b^
Days—SCT to neutrophil engraftment			
*n*	22	39	
Mean (SD)	14 (6)	14 (5)	
Median (Min; Max)	12 (9; 31)	13 (1; 34)	0.51^b^
LOS—SCT hospital stay			
*n*	26	43	
Mean (SD)	28 (11)	30 (11)	
Median (Min; Max)	26 (6; 60)	27 (21; 65)	0.76^b^

^b^Wilcoxon Rank sum

^c^Chi square

Considering only very poor mobilizers (CD34+ count ≤ 10/µl), 7 out of 15 patients (47%) in the pre-plerixafor period and 33/40 patients (83%) in the plerixafor period proceeded to transplantation (*p* = 0.02). Again, there was no difference between platelet/neutrophil engraftment rates and time to engraftment in those patients proceeding to transplantation (Table 5).

**Table 5:** Transplantation and engraftment in patients with CD34+ count < 10 cells/µl

	Pre-plerixafor era (*n* = 15)	Plerixafor era (*n* = 40)	*p*-value
Patients proceeding to SCT; *n* (%)	7/15 (47)	33/40 (83)	0.02^d^
SCT patients achieving platelet engraftment	4/7 (57)	20/33 (61)	1.00^d^
SCT patients achieving neutrophil engraftment	6/7 (86)	29/33 (88)	1.00^d^
Days—Apheresis to SCT			
* n*	4	33	
Mean (SD)	73 (61)	61 (50)	
Median (Min; Max)	53 (32; 208)	42 (28; 286)	0.29^b^
Days—SCT to platelet engraftment			
*n*	4	33	
Mean (SD)	20 (17)	16 (12)	
Median (Min; Max)	13 (10; 45)	14 (1; 58)	0.94^b^
Days—SCT to neutrophil engraftment			
* n*	6	29	
Mean (SD)	14 (8)	14 (4)	
Median (Min; Max)	11 (9; 31)	13 (9; 24)	0.38^b^
LOS—SCT hospital stay			
*n*	9	32	
Mean (SD)	29 (17)	29 (10)	
Median (Min; Max)	24 (6;60)	28 (21; 65)	0.42^b^

^b^Wilcoxon rank sum

^d^Fisher’s exact

However, more patients in the plerixafor period received transfusions of platelets and red blood cells. These differences were statistically significant (Table SI 2). The average number of platelet transfusions per patient was higher in the plerixafor period in comparison to the pre-plerixafor era.

## Discussion

**Fig. 1:**
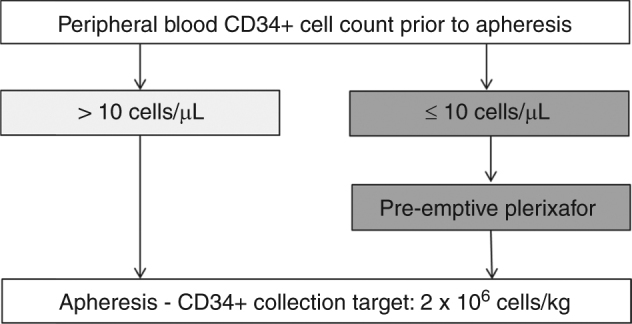
Schematic representation of current mobilization practice with plerixafor in Germany

Plerixafor, a CXCR4 inhibitor increases the amount of circulating stem cells several folds when given in combination with conventional mobilization regimens. In Europe, plerixafor is approved in combination with G-CSF with or without chemotherapy in patients with multiple myeloma or lymphoma who are candidates for ASCT but whose cells mobilize poorly. The definition of poor mobilizers remains however vague and reflects the difficulty in defining the exact patient population for whom plerixafor may be considered cost-effective. The recent European position statement regarding autologous stem cell mobilization recommends the use of plerixafor in a dynamic way in patients with CD34+ cell count between 10–20 CD34+ cells/µl depending on patient characteristics and treatment history. In Germany and during the inclusion period of this study until 2014, most centers used plerixafor pre-emptively only in patients with a CD34+ count ≤ 10 cells/µl as schematically shown in Figure 1. Whether this management approach is the most cost-effective option remains to be determined.

In a recent, multinational, non-interventional study the impact of plerixafor on poor mobilizers (CD34+ cell level < 20/µl) was analyzed by comparing apheresis outcomes in the period prior and after introduction of plerixafor to the market. Overall, plerixafor reduced the average number of apheresis sessions per patient, and the average time spent on apheresis in poor mobilizers. However, country-specific differences were observed, with plerixafor having a lower impact on apheresis time and costs in Germany compared to the other sites. The goal of this study was therefore to re-analyze German specific data.

There was a non-significant decrease in the average number of apheresis sessions from 1.9 to 1.6 sessions per patient, leading to a reduction in apheresis costs of €866 and a small decrease in total apheresis time. These findings are less marked than those obtained from non-German sites or those reported in other cost-effectiveness studies with plerixafor [18].

There are several possible reasons for a smaller effect size seen in the German setting: First, there might be differences in patient characteristics during the two time-periods. In the pre-plerixafor era, the initial CD34+ cell count was significantly higher than that observed in the plerixafor era (11.7/µl vs 7.1/µl; *p* < 0.001). The proportion of poor mobilizers with CD34+ count ≤ 10/µl was smaller in the pre-plerixafor era as compared to the plerixafor era: 15/39 (38%) vs 40/51 (78%) patients, respectively. It is hypothesized that prior to the introduction of plerixafor, hematologists were reluctant to carry out apheresis on patients with CD34+ count ≤ 10/µl because of the greater risk of collection failure. With the introduction of plerixafor, more of these patients were considered for apheresis leading to a population which was more difficult to mobilize. To account for differences in patient characteristics between the two eras, a subgroup analysis was carried out in patients with CD34+ count ≤ 10 cells/µl. In this population, the number of apheresis sessions per patient significantly decreased from 2.1 sessions in the pre-plerixafor era to 1.6 sessions in the plerixafor era (*p* < 0.01). At the same time, the total time spent on apheresis decreased from 429 min to 338 min (*p* = 0.04) and the CD34+ yield after first apheresis increased from 1.5 to 2.6 (*p* < 0.01). Cost associated with apheresis decreased from €6246 to €4758 (*p* < 0.01).

Second, hospitals in Germany are reimbursed per patient, not per apheresis session versus French hospitals being reimbursed per apheresis session. German centers have little financial incentive to carry out additional apheresis sessions to obtain higher CD34+ cell yields, as long as sufficient stem cells have been collected to proceed to transplantation. Therefore, the number of apheresis sessions per patient was already relatively low in Germany during the pre-plerixafor era (1.9 sessions per patient in Germany vs. 2.4 sessions in France)[17].

Third, mobilization efficacy might be different in German patients as compared to patients from other countries due to population characteristics and chemotherapy regimens. In a retrospective study conducted in Germany prior to the introduction of plerixafor, Wuchter et al found that NHL patients identified as poor mobilizers, all of those with CD34+ cell count between 11–19/µl (i.e., “borderline” poor mobilizers) collected sufficient hematopoietic stem cells (2.0 × 10^6^ cells/kg). On the other hand, among patients with a CD34+ cell count between 6–10/µl and less than 5/µl only 65% and 35% achieved their collection target [7].

A threshold of 20 cells/µl for circulating CD34+ cells has initially been proposed to define poor mobilizers based on data by Pusic et al who showed a CD34+ cell count of at least 20 cells/µl was predictive of successful day 1 apheresis [16]. Other studies have found similar values, but a universal cut-off value has not been established as it depends on the mobilization regimen, treatment goals, and patient characteristics. Our data suggest that in Germany plerixafor significantly reduced costs and time spent on apheresis in patients with initial CD34+ count ≤ 10 cells/µl justifying the current management approach (Fig. 1). In comparison, data specific to France from the same study showed a significant reduction in apheresis costs and total time for the general population of poor mobilizers with CD34+ < 20 cells/µl [17].

Even though not a primary endpoint, it should be highlighted that in the pre-plerixafor era 24/39 poor mobilizers (62%) proceeded to transplantation. After the introduction of plerixafor, 44/51 poor mobilizers (86%) could proceed to transplantation (*p* < 0.01). This difference was even more marked in poor mobilizers with initial CD34+ count ≤ 10 cells/µl where 7/15 (47%) of patients in the pre-plerixafor era and 33/40 (83%) of patients in the plerixafor proceeded to transplantation respectively (*p* = 0.02). This may in part be explained by an increase in the proportion of patients reaching at least 2.0 × 10^6^ cells/kg; however, such an increase was only observed in patients with CD34+ count ≤ 10 cells/µl, (67 vs 90% in the pre-plerixafor and plerixafor era, respectively) and not in patients with initial CD34+ cell count > 10 cells/µl (100 vs 91% in the pre-plerixafor and plerixafor era, respectively). Failure to proceed to transplantation might therefore be linked to factors other than the mobilization regimen. Rates of successful engraftment and time to engraftment did not differ between the two time periods, in agreement with other reports which showed that plerixafor didn’t impact engraftment rates as long as the target CD34+ cell yield could be achieved [7, 19, 20]. However, the need for transfusions was higher in the plerixafor era as compared to the pre-plerixafor era. There is no clear explanation to this finding, but one hypothesis is that patients proceeding to transplantation were in a less favorable overall clinical status in the plerixafor era as compared to those in the pre-plerixafor era. In comparison, in France and Italy, the proportion of patients proceeding to transplantation remained constant with no large differences in transfusion requirements observed [SI, Fig. 1, SI Tables 3 & 4].

This study has several limitations: a narrow perspective was chosen with the main outcomes being time spent on apheresis and costs associated with apheresis. Costs for resources spent on mobilization and mobilization regimens were not included, nor were costs associated with transplantation and post-transplantation care considered. A direct comparison to other cost-effectiveness studies covering the entire ASCT process is therefore not possible. An additional limitation regards the relatively small number of patients included. In the subgroup of patients with initial CD34+ cell count of ≤10 cells/µl comprised 15 and 40 patients in the pre-plerixafor and plerixafor era, respectively. It should be noted that in this study, poor mobilizers not undergoing apheresis were excluded and therefore, the impact of plerixafor on all poor mobilizers cannot be evaluated. However, our results suggest that more patients with initial CD34+ cell count of ≤10 cells/µl were selected for HDC and ASCT upon introduction of plerixafor. In the subgroup of patients with initial CD34+ cell count > 10 cells/µl, no statistically significant differences between the two eras were observed for any of the study endpoints (SI Table 5). The number of patients in this subgroup was however limited, preventing robust conclusions and suggesting that only few patients with initial CD34+ cell count > 10 cells/µl received plerixafor pre-emptively, in line with the current practice in Germany (Fig. 1).

In conclusion, with the introduction of plerixafor in Germany, more patients with initial CD34+ cell count ≤ 10 cells/µl were considered for apheresis and subsequent transplantation. Time spent on apheresis and costs related to apheresis were significantly reduced in poor mobilizers with initial CD34+ cell count ≤ 10 cells/µl while there was no significant reduction in time and effort spent on apheresis in the overall population of poor mobilizers. A more modest effect of plerixafor on apheresis outcomes in Germany compared to other European countries might be due to reimbursement policies, patient characteristics, clinical practice or a mixture of different factors. Regardless of initial CD34+ cell count, more patients proceeded to transplantation in the plerixafor era as compared to the pre-plerixafor era. The results of this study consolidate the use of pre-emptive plerixafor in patients with initial CD34+ count ≤ 10 cells/µl. The impact of plerixafor on the rate of patients proceeding to transplantation deserves further study.

## Electronic supplementary material

Supplemental Material
