# Supplementary material for: Plerixafor in non-Hodgkin’s lymphoma patients: a German analysis of time, effort and costs
Source: Bone Marrow Transplant. 2018 May 24;54(1):123–9. doi: 10.1038/s41409-018-0228-z (PMC6320344; doi:10.1038/s41409-018-0228-z)
Supplement: Supplementary file 1 — Supplemental Material [file 41409_2018_228_MOESM1_ESM.docx]

**Supplementary information**

SI Table 1 : Micro-costing per apheresis session (€)

|  | Cost (€) |
| --- | --- |
| Pre-apheresis Laboratory | 427 |
| Apheresis | 1369 |
| Manipulation, storage, and thawing | 1132 |
| Total | 2928 |

SI Table 2 : Transfusions - Germany

|  | Pre-plerixafor era (n=39) | | Plerixafor era (n=51) | | p-value |
| --- | --- | --- | --- | --- | --- |
| Patients receiving platelets ; n (%) | 23 | 59% | 43 | 84% | 0.01 (c) |
| Patients receiving PRBCs; n(%) | 20 | 51% | 37 | 73% | 0.04 (c) |
| Platelet Transfusions per Patient |  |  |  |  |  |
| Mean (SD) | 2.3 | 3.1 | 3.9 | 4.0 |  |
| Median (Q1-Q3) | 1 | 0-4 | 3 | 1-5 |  |
| Range (min-max) |  | 0-15 |  | 0-18 | 0.01 (b) |
| PRBC Transfusion per Patient |  |  |  |  |  |
| Mean (SD) | 0.9 | 1.1 | 1.4 | 1.4 |  |
| Median (Q1-Q3) | 0 | 0-2 | 1 | 0-2 |  |
| Range (min-max) |  | 0-4 |  | 0-6 | 0.08 (b) |

(b) Wilcoxon Rank Sum

(c) Chi Square


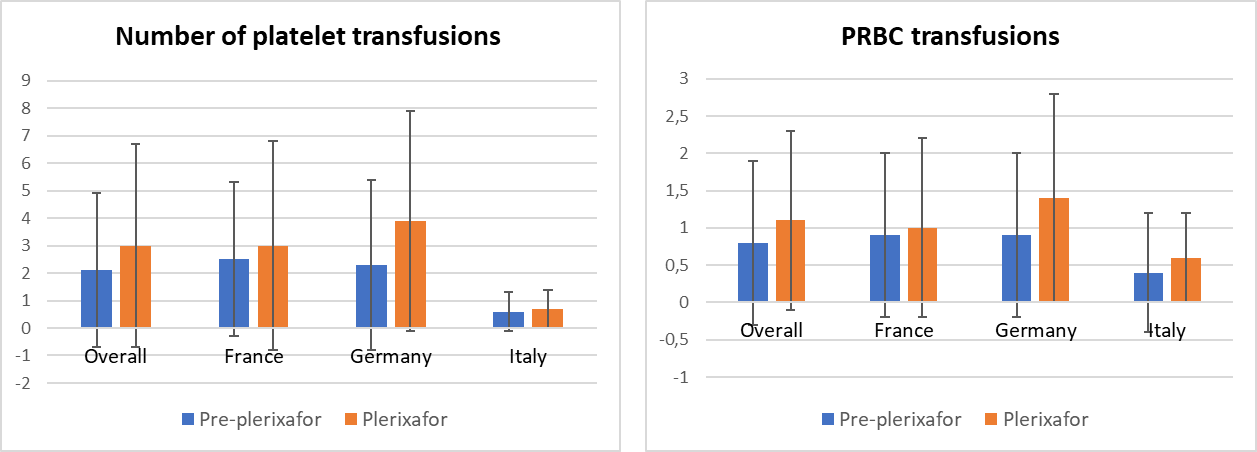


SI Figure 1 : Transfusion requirements in the pre-and plerixafor era analyzed by site ; Standard deviations are depicted as vertical black lines.

SI Table 3 : Transfusions - France

|  | Pre-plerixafor era (n=59) | | Plerixafor era (n=61) | | p-value |
| --- | --- | --- | --- | --- | --- |
| Patients receiving platelets ; n (%) | 39 | 66% | 41 | 65% | 0.91 (c) |
| Patients receiving PRBCs; n(%) | 31 | 53% | 36 | 53% | 0.61 (c) |
| Platelet Transfusions per Patient |  |  |  |  |  |
| Mean (SD) | 2.5 | 2.8 | 3.0 | 3.8 |  |
| Median (Q1-Q3) | 2 | 0-4 | 1 | 0-5 |  |
| Range (min-max) |  | 0-13 |  | 0-15 | 0.96 (b) |
| PRBC Transfusion per Patient |  |  |  |  |  |
| Mean (SD) | 0.9 | 1.1 | 1.0 | 1.2 |  |
| Median (Q1-Q3) | 1 | 0-2 | 1 | 0-2 |  |
| Range (min-max) |  | 0-4 |  | 0-5 | 0.64 (b) |

(b) Wilcoxon Rank Sum

(c) Chi Square

SI Table 4 : Transfusions - Italy

|  | Pre-plerixafor era (n=20) | | Plerixafor era (n=20) | | p-value |
| --- | --- | --- | --- | --- | --- |
| Patients receiving platelets ; n (%) | 9 | 45% | 12 | 60% | 0.34 (c) |
| Patients receiving PRBCs; n(%) | 4 | 20% | 10 | 58% | 0.05 (c) |
| Platelet Transfusions per Patient |  |  |  |  |  |
| Mean (SD) | 0.6 | 0.7 | 0.7 | 0.7 |  |
| Median (Q1-Q3) | 0 | 0-1 | 1 | 0-1 |  |
| Range (min-max) |  | 0-2 |  | 0-3 | 0.49 (b) |
| PRBC Transfusion per Patient |  |  |  |  |  |
| Mean (SD) | 0.3 | 0.6 | 0.6 | 0.6 |  |
| Median (Q1-Q3) | 0 | 0-0 | 1 | 0-1 |  |
| Range (min-max) |  | 0-2 |  | 0-2 | 0.07 (b) |

(b) Wilcoxon Rank Sum

(c) Chi Square

SI Table 5 : Apheresis activities in patients with CD34+ count > 10 cells/µl

|  | **Pre-plerixafor era (n=24)** | | **Plerixafor era (n=11)** | | **p-value** |
| --- | --- | --- | --- | --- | --- |
| Initial peripheral CD34+ (cells/µl) |  | |  | |  |
| Mean (SD) | 15.5 | (3.2) | 14.3 | (2.9) |  |
| Median (Min; Max) | 16.0 | (10.3;20.0) | 14.0 | (10.3; 19.8) | 0.25 (b) |
| Number of apheresis sessions |  |  |  |  |  |
| Mean (SD) | 1.8 | (0.9) | 1.6 | (0.7) |  |
| Median (Min; Max) | 1.5 | (1.0; 4.0) | 2.0 | (1.0; 3.0) | 0.82 (b) |
| Estimated apheresis cost (€) |  |  |  |  |  |
| Mean (SD) | 5246 | (2728) | 4791 | (1974) |  |
| Median (Min; Max) | 4392 | (2928; 11712) | 5856 | (2928; 8784) | 0.82 (b) |
| Total apheresis blood volume (l) |  |  |  |  |  |
| Mean (SD) | 23.6 | (13.7) | 22.1 | (12.5) |  |
| Median (Min; Max) | 16.9 | (9.9; 51.2) | 21.0 | (11.4; 54.0) | 0.93 (b) |
| Total minutes of apheresis |  |  |  |  |  |
| Mean (SD) | 357 | (169) | 306 | (1) |  |
| Median (Min; Max) | 308 | (135; 1000) | 265 | (125; 495) | 0.44 (b) |
| CD34+ cells, total (x10^6^ cells/kg) |  |  |  |  |  |
| Mean (SD) | 6.9 | (3.7) | 5.2 | (3.9) |  |
| Median (Min; Max) | 5.7 | (2.1; 16.7) | 3.7 | (1.8; 13.8) | 0.08 (b) |
| CD34+ cells, first apheresis (x10^6^ cells/kg) |  |  |  |  |  |
| Mean (SD) | 4.8 | (3.6) | 4.1 | (4.5) |  |
| Median (Min; Max) | 4.0 | (1.0; 12.2) | 1.5 | (0.9; 13.8) | 0.36 (b) |

(b) Wilcoxon Rank Sum
